# Galectin-1 expression imprints a neurovascular phenotype in proliferative retinopathies and delineates responses to anti-VEGF

**DOI:** 10.18632/oncotarget.17129

**Published:** 2017-04-16

**Authors:** Magali E. Ridano, Paula V. Subirada, María C. Paz, Valeria E. Lorenc, Juan C. Stupirski, Ana L. Gramajo, José D. Luna, Diego O. Croci, Gabriel A. Rabinovich, María C. Sánchez

**Affiliations:** ^1^ Centro de Investigaciones en Bioquímica Clínica e Inmunología (CIBICI), Consejo Nacional de Investigaciones Científicas y Técnicas (CONICET), Departamento de Bioquímica Clínica, Facultad de Ciencias Químicas, Universidad Nacional de Córdoba, Córdoba, Argentina; ^2^ Laboratorio de Inmunopatología, Instituto de Biología y Medicina Experimental (IBYME), CONICET, Buenos Aires, Argentina; ^3^ Centro Privado de Ojos Romagosa-Fundación VER, Córdoba, Argentina; ^4^ Departamento de Química Biológica, Facultad de Ciencias Exactas y Naturales, Universidad de Buenos Aires, Buenos Aires, Argentina; ^5^ Department of Ophthalmology, The Johns Hopkins School of Medicine, Baltimore, MD, United States; ^6^ Laboratorio de Inmunopatología, Instituto de Histología y Embriología de Mendoza (IHEM), CONICET, Universidad Nacional de Cuyo, Facultad de Ciencias Exactas y Naturales (FCEN), Mendoza, Argentina

**Keywords:** galectin-1, neovascularization, neurodegeneration, retinopathies, vascular endothelial growth factor

## Abstract

Neovascular retinopathies are leading causes of irreversible blindness. Although vascular endothelial growth factor (VEGF) inhibitors have been established as the mainstay of current treatment, clinical management of these diseases is still limited. As retinal impairment involves abnormal neovascularization and neuronal degeneration, we evaluated here the involvement of galectin-1 in vascular and non-vascular alterations associated with retinopathies, using the oxygen-induced retinopathy (OIR) model. Postnatal day 17 OIR mouse retinas showed the highest neovascular profile and exhibited neuro-glial injury as well as retinal functional loss, which persisted until P26 OIR. Concomitant to VEGF up-regulation, galectin-1 was highly expressed in P17 OIR retinas and it was mainly localized in neovascular tufts. In addition, OIR induced remodelling of cell surface glycophenotype leading to exposure of galectin-1-specific glycan epitopes. Whereas VEGF returned to baseline levels at P26, increased galectin-1 expression persisted until this time period. Remarkably, although anti-VEGF treatment in P17 OIR improved retinal vascularization, neither galectin-1 expression nor non-vascular and functional alterations were attenuated. However, this functional defect was partially prevented in galectin-1-deficient (*Lgals1*−/−) OIR mice, suggesting the importance of targeting both VEGF and galectin-1 as non-redundant independent pathways. Supporting the clinical relevance of these findings, we found increased levels of galectin-1 in aqueous humor from patients with proliferative diabetic retinopathy and neovascular glaucoma. Thus, using an OIR model and human samples, we identified a role for galectin-1 accompanying vascular and non-vascular retinal alterations in neovascular retinopathies.

## INTRODUCTION

Proliferative neovascular retinopathies are major leading causes of blindness in industrialized countries [1]. Vision loss in retinopathy of prematurity and proliferative diabetic retinopathy are caused by several growth factors [2, 3], including vascular endothelial growth factor-A (VEGF-A), the most potent cytokine that mediates ischemia-induced retinal neovascularization (NV) in ocular pathologies [4, 5]. VEGF-A exerts its effects *via* activation of various signaling events, including tyrosine phosphorylation of its receptors VEGFR1 (Flt-1), VEGFR2 (KDR/Flk-1), and VEGFR3 (Flt-4) and their downstream effectors on endothelial cells (ECs) [6].

Accumulating evidences, stemming from experimental models and clinical studies have provided insights into the mechanisms of vascular injury leading to pathological vitreoretinal NV, leading to discovery and implementation of ocular anti-VEGF therapies [7]. Bevacizumab (Avastin^®^; Genentech, San Francisco, CA), ranibizumab (Lucentis^®^; Genentech, San Francisco, CA), and aflibercept (Eylea^®^; Regeneron Pharmaceuticals, Tarrytown, NY) are the most widely used anti-VEGF drugs administered by intravitreal injection in ocular pathologies. However, the clinical benefit conferred by these therapies is variable and the results have not always been successful in preserving retinal function [2].

In addition to the vascular component of neovascular retinopathies, several studies have shown that local neurons and glial cells are also affected in the ischemic retina and their dysfunction may exacerbate the aberrant angiogenic phenotype and contribute to progression of the disease [8–10]. In fact, Müller glial cells contribute to the development of pathological NV [11–14] and neurodegeneration plays a significant role in microvascular impairment [15], indicating that dual targeting of both vascular and neural compartments may represent an effective strategy for treating this multifactorial disease. Thus, a next generation of pharmacological agents for neovascular retinopathies should prevent or improve both pathological NV as well as neuron and glial alterations. In this regard, the mouse model of oxygen-induced retinopathy (OIR) has been instrumental for understanding the mechanisms underlying retinal pathogenesis as well for developing groundbreaking therapeutics offering an opportunity to examine the role of hypoxia and hypoxia-regulated genes in the pathogenesis of retinal NV, neuroinflammation, oxidative stress and neurovascular cross-talk [16].

Galectin-1 (Gal1), a member of a highly conserved family of animal lectins, has been implicated in angiogenesis-related disorders including cancer and endometriosis through hypoxia-inducible factor 1 (HIF-1α)- and VEGF-independent pathways [17–22]. Gal1 recognizes poly-N-acetyllactosamine (*LacNAc*) structures in *N*- and *O*- glycans present on a selected repertoire of glycosylated receptors [23] and promotes tumor progression through mechanisms involving promotion of tumor vascularization, immunosuppression and metastasis [24–27]. Interestingly, Gal1 is upregulated by hypoxia [17, 28] and controls ECs signaling [17] and VEGFR2 trafficking [29]. Moreover, we found that glycosylation-dependent programs mediated by Gal1 association to VEGFR2 promote VEGF-like signaling and preserve angiogenesis in anti-VEGF refractory tumors [30].

In this study we evaluated the contribution of Gal1 to functional impairment during the pathogenesis of experimental neovascular retinopathies and its regulated expression during the development of vascular and non-vascular alterations using the OIR model in mice treated or not with anti-VEGF mAb. Moreover, we validated the clinical relevance of these observations in aqueous humor of diabetic patients with neovascular retinopathies.

## RESULTS

### Increased expression of Gal1 during retinal impairment in the OIR model

**Figure 1:**
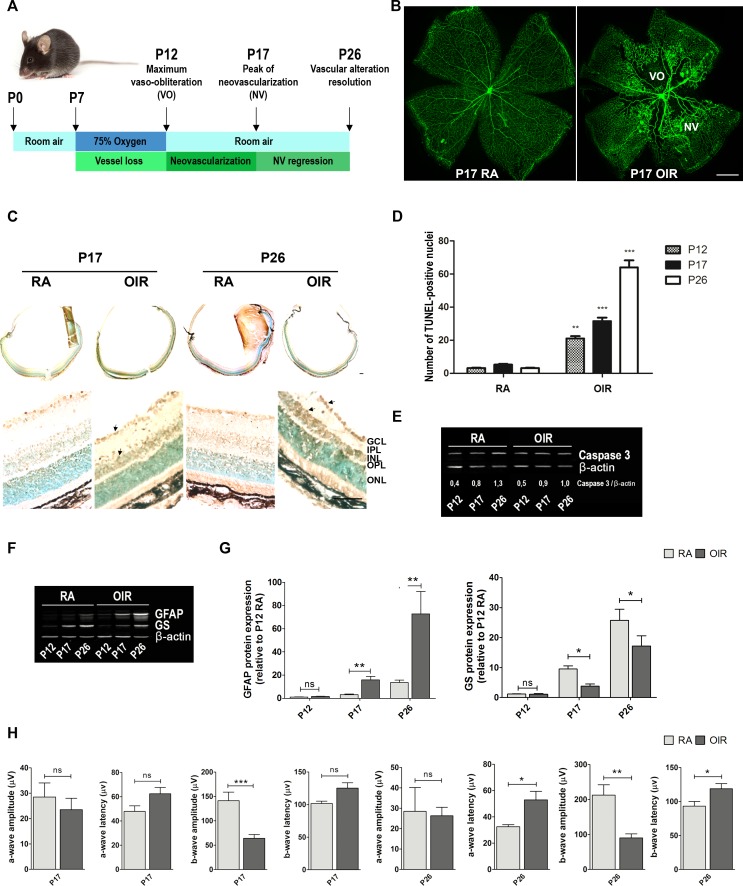
Neovascularization, neurodegeneration, glial activation and functional loss in OIR mouse retinas **A**. Scheme representing the OIR mouse model with hallmark time points during experimental disease development. Neonatal mice and their nursing mother are kept in room air from birth to P7 and normal vascular development ensues. At P7, they are exposed to 75% oxygen, which inhibits retinal vessel growth and causes significant VO. Mice are returned to room air at P12; the avascular retina becomes hypoxic, eliciting both normal vessel regrowth and pathological neovascular response. NV reaches its maximum at P17. Shortly thereafter, it spontaneously regresses and the vascular alterations resolve by P25. This scheme is adapted from Connor KM *et al*. Nat Protoc. 2009;4:1565-1573. **B**. Representative images of whole mount retinas at P17 showing GSA-IB4 vascular staining in RA and OIR mice. Areas with VO and NV are indicated. Scale bar: 100 μm. **C**. Representative TUNEL cryosections labeling of RA and OIR mice, at P17 and P26. Scale bar: 50 μm. Abbreviations: GCL, ganglion cell layer; IPL, inner plexiform layer; INL, inner nuclear layer; OPL, outer plexiform layer; ONL, outer nuclear layer. Arrows are indicating TUNEL-positive nuclei. **D**. Quantification of the TUNEL-positive cells between both groups at P17 and P26 are shown. **E**. and **F**. Representative Western blot of total caspase-3, GFAP and GS from neural retinal extracts of RA and OIR mice at P12, P17 and P26. β-actin is shown as a loading control. **G**. Levels of GFAP and GS were quantified by densitometry and normalized by β-actin. Graph shows results from four independent experiments. **H**. Amplitudes and latencies of a- and b-waves from scotopic ERG were recorded at P17 and P26 in RA and OIR mice. Data show the average of responses over both eyes, in five mice per condition. Data are presented as mean ± SEM. ns, non-significant, *p < 0.05, **p < 0.01, *** *p* < 0.001.

In order to explore vascular and non-vascular alterations associated to neovascular retinopathies and the consequent functional retinal loss, we first analyzed NV, neurodegeneration and electroretinogram responses in the OIR mouse model. Figure 1A summarizes the most important functional hallmarks featuring different individual time periods in this animal model [31]. At P12 OIR, a pronounced vaso-obliteration (VO) effect generated by hyperoxia was observed. Moreover, during the relative hypoxic period, animals developed extensive retinal NV (peak is reached at P17) and from P17 onward NV declined, until P25 where almost no VO or NV remained visible as described [32].

GSA-IB4 lectin-labeled blood vessels in flat-mount retinas showed, at P17 OIR, a central zone of VO in addition to the characteristic vitreoretinal neovascular tufts, which were not observed in P17 room air controls (RA) (Figure 1B). Moreover, TUNEL-positive nuclei were detected in the ganglion cell layer (GCL), within the inner nuclear layer (INL; mainly neurons such as bipolar and amacrine cells), as well as in outer nuclear layer (ONL; photoreceptors) in OIR retinas (Figure 1C). Quantitative analysis showed that TUNEL-positive cells were significantly higher in P12 and P17 OIR compared to control mice (RA), with a significant increase at day P26 OIR (Figure 1D). Besides, Western blot analysis of retinas from RA and OIR mice revealed decreased levels of total caspase-3 protein in P26 OIR (Figure 1E), suggesting an increased expression of its cleaved form [33]. In addition, expression of glial fibrillary acidic protein (GFAP) substantially increased in P17 and even more in P26 OIR respect to RA mouse retinas (Figures 1F and 1G), in accordance with activation of Müller glial cells in the mouse model. Moreover, we observed lower expression of glutamine synthase (GS) in both P17 and P26 OIR respect to RA mouse retinas suggesting impairment in glutamate detoxification (Figures 1F and 1G).

Next, we analyzed the functional status of retinas by scotopic electroretinography (ERG). Amplitude and latency of electroretinogram a- and b- waves at P17 and P26 of RA and OIR mice were registered. The b-wave amplitude was significantly reduced in OIR compared to RA mice. Also, a- and b- wave latencies were significantly increased in P26 OIR, and a tendency toward larger latencies was observed in P17 OIR mice respect to RA (Figure 1H), indicating that retinal dysfunction was progressive. These results show that, although retinal NV and VO were completely resolved in this mouse model, neuronal damage, glial activation and functional loss persisted until P26 OIR.

**Figure 2:**
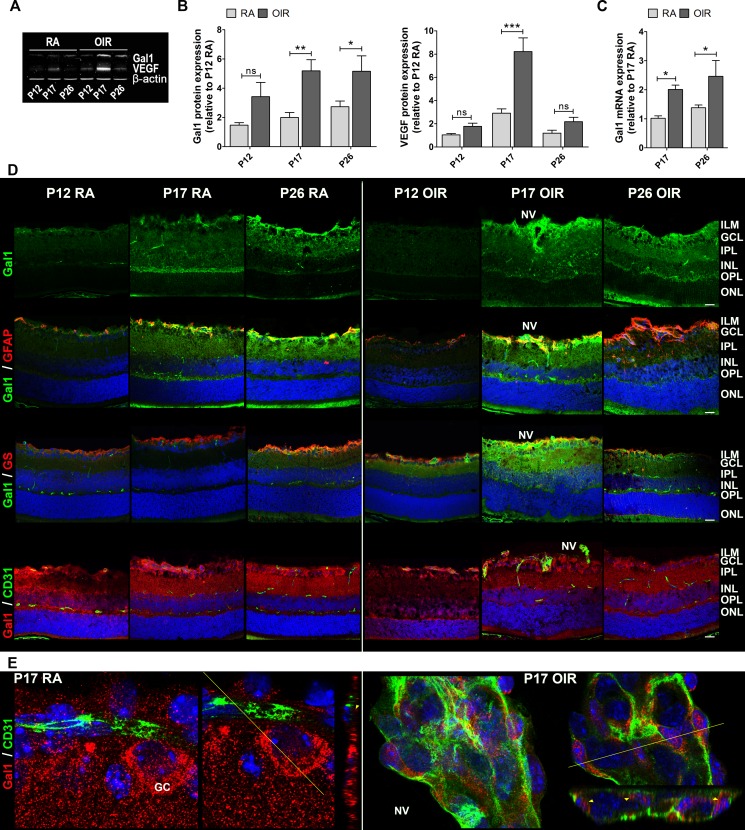
Gal1 expression and localization in RA and OIR mouse retinas **A**. Representative Western blot of Gal1 and VEGF from neural retina extracts of RA and OIR mice at P12, P17 and P26. β-actin is shown as a loading control. **B**. Levels of Gal1 and VEGF were quantified by densitometry and normalized by β-actin. Results of at least four independent experiments are shown. **C**. Gal1 mRNA was quantified by qRT-PCR in neurosensory retinas of P17 and P26 OIR mice or RA (control) conditions. Results were normalized to β-actin and expressed according to the 2^−ΔΔCt^ method using as calibrator the mRNA level obtained from P17 RA mouse retinas. Data are presented as mean ± SEM. ns, non-significant, *p < 0.05, **p < 0.01, *** *p* < 0.001. **D**. Representative immunofluorescence analysis of Gal1 (green) in cryosections of RA and OIR mouse retinas at P12, P17 and P26 (first panel). Double labeling using a mouse monoclonal antibody for Gal1 (green) and an astrocyte and activated MGC marker, anti-GFAP (red) (second panel), or a MGC-specific marker, anti-GS (red) (third panel) and a rabbit polyclonal anti-Gal1 (red) in combination with an endothelial cell marker, anti-CD31 (green) (fourth panel). Cell nuclei were counterstained with Hoechst 33258 (blue). Scale bar: 25 μm. **E**. High magnification confocal micrograph of the representative P17 RA and OIR retinas showing a typical vessel (left panel) and a neovascular tuft (right panel). Images were taken with silicon 60X objective in the best confocal resolution condition and spatial deconvolution was done with the Huygeng Professional software. X, Y and Z reconstruction images (left) and a representative image with a line indicating the zone of Z profile (right) are shown. Yellow arrowheads indicate Gal1 distribution in RA and in OIR endothelial cells. Abbreviations: ILM, inner limiting membrane; GCL, ganglion cell layer; IPL, inner plexiform layer; INL, inner nuclear layer; OPL, outer plexiform layer; ONL, outer nuclear layer; NV, neovascularization; GC: ganglion cell.

Seeking for VEGF-independent pathways that could modulate neovascular retinopathies outcome, we explored the relevance of Gal1 in the pathogenesis of this retinal disease using the OIR mouse model. We measured Gal1 and VEGF expression by Western blot in RA *vs*. OIR mice at critical time points (Figure 2A). Interestingly, Gal1 expression increased during retinal development in RA, being statistically higher in P26, but not in P17 respect to P12 RA (data not shown). Quantitative analysis revealed that Gal1 protein levels were significantly higher in retinas of P17 and P26 OIR compared to RA mice; while, as expected, VEGF protein levels peaked at P17 returning to baseline levels at P26 (Figure 2B). In addition, Gal1 mRNA levels significantly increased in the neurosensory retina at P17 and P26, suggesting that the OIR condition induces Gal1 expression in the retina (Figure 2C). Then, we analyzed Gal1 localization in retinas of both normal and pathological groups (RA and OIR) by immunofluorescence assays. Confocal microscopy analysis revealed that Gal1 was localized within the inner layers of the retina, mainly at the inner limitant membrane (ILM) and GCL under retinal development; however, in the peak of the NV of the OIR mouse model (P17), expression of this lectin was most prominent in the most highly vascularized area (Figure 2D, upper panel). Higher Gal1 expression, occurred at the same time as the neural retina modified its structure in response to hypoxia and the Müller glial cells initiated an activation program as confirmed by GFAP positive staining (Figure 2D, second panel). To determine the cellular sources contributing to Gal1 expression, immunofluorescence was performed using a combination of two different anti-Gal1 antibodies (mouse monoclonal and rabbit polyclonal antibodies) along with anti-GFAP (rabbit polyclonal), anti-GS (rabbit polyclonal) or anti-CD31 (PCAM-1) (mouse monoclonal) antibodies and colocalization studies were carried out. Increased colocalization of Gal1 and GFAP was observed by confocal microscopy at P17 and P26 RA mice retinas, suggesting that Gal1 is present in astrocytes and in the endfeet of activated Müller glial cells in OIR, mainly at P17 (Figure 2D, second panel). GS and Gal1 colocalization in P17 OIR mouse retinas strengthened this observation (Figure 2D, third panel). Then, we examined whether CD31^+^ cells were also positive for Gal1 given that CD31 makes up a large portion of endothelial cell intercellular junctions. Results demonstrated that Gal1 did not colocalize with CD31 at any of the times evaluated; however, a relationship between signals was detected (Figure 2D, bottom panel). At higher magnification, Gal1 was detected surrounding CD31^+^ cells in P17 RA mouse retinas (normal vessels); nevertheless, during the peak of the NV (P17 OIR), Gal1 was also found in neovascular tufts within CD31^+^ cells (Figure 2E). Thus, up-regulation of Gal1 during OIR and its localization in areas with high NV as well as inside neovascular tufts, suggest a role for this endogenous lectin in the pathogenesis of experimental neovascular retinopathies.

### Differential glycosylation profile of mouse retinas in RA and OIR

**Figure 3:**
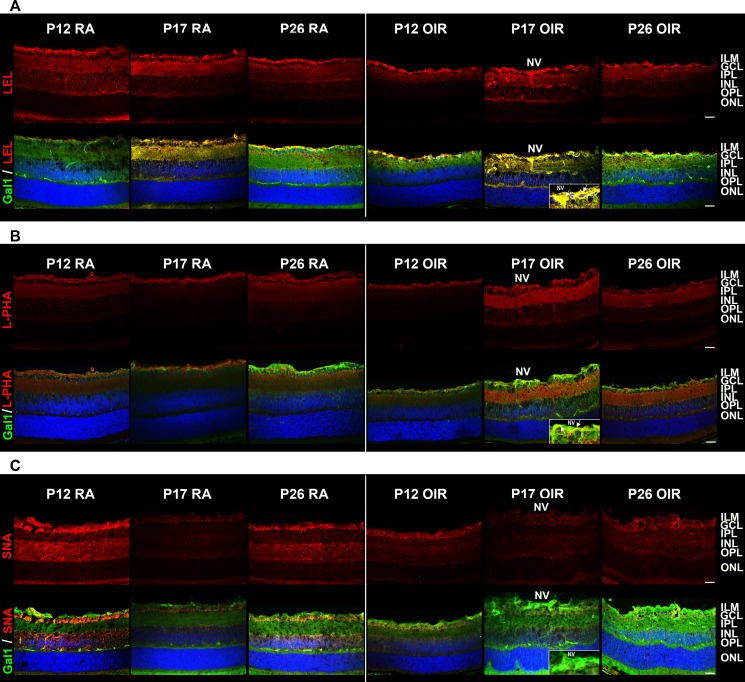
Glycosylation profile of RA and OIR mouse retinas Representative staining of LEL **A**., L-PHA **B**. and SNA **C**. in red (upper panel) and its combination with anti-Gal1 in green (bottom panel) in cryosections of P12, P17 and P26 RA and OIR retinas. Cell nuclei were counterstained with Hoechst 33258 (blue). Arrows indicate areas of colocalization in neovessels (yellow). All the experiments were performed in triplicate and are representative of three independent experiments. Abbreviations: ILM, inner limiting membrane; GCL, ganglion cell layer; IPL, inner plexiform layer; INL, inner nuclear layer; OPL, outer plexiform layer; ONL, outer nuclear layer; NV, neovascularization. Scale bar: 25 μm.

As Gal1 modulates cellular programs by recognizing poly-*LacNAc* structures in *N*- and *O*- glycans present on a selected repertoire of glycosylated receptors [24], we examined the ‘glycosylation signature’ of mouse retinas during development (RA) and following exposure to hypoxic stimuli (OIR), using a panel of biotinylated lectins that recognize specific glycan structures particularly those that are relevant for Gal1 binding [30]. Staining by: a) *Lycopersicon esculentum* lectin (LEL) which recognizes poly-LacNAc structures (Figure 3A, upper panel); b) *L-phytohemagglutinin* (L-PHA) which binds to β1-6-N-acetylglucosamine (β1-6GlcNAc)-branched complex *N*-glycans, which are the preferred intermediates for LacNAc extension (Figure 3B, upper panel); and c) *Sambucus nigra* agglutinin (SNA) (Figure 3C, upper panel) which recognizes α,6-linked sialic acid, revealed considerable changes in the glycosylation pattern of mouse retina during development and when exposed to hypoxic conditions. Notably, colocalization of LEL with Gal1 was almost complete in retinas at P17 in both RA and OIR (Figure 3A, bottom panel). However, in OIR, L-PHA reactivity was much more intense than control conditions and the colocalization with Gal1 was greater in areas with NV at P17 in OIR respect to RA retinas (Figure 3B, bottom panel), suggesting that Gal1 may preferentially associate with elongated LacNAc structures present in complex branched N-glycans. Finally, SNA reactivity predominated in GCL and INL, was markedly decreased in P17 and P26 OIR and did not colocalize with Gal1 (Figure 3C, bottom panel), consistent with the ability of α2,6-linked sialic acid to mask Gal1-specific glyco-epitopes [32]. These results indicate that OIR conditions alter the ‘glycosylation signature’ of the normal retina and suggest that areas with NV display a glycosylation pattern permissive for Gal1 binding as evidenced by increased amount of β1-6GlcNAc-branched complex *N*-glycans, abundant *LacNAc* structures and reduced α2,6 sialylation of cell surface glycoproteins respect to RA retinas.

### Anti-VEGF treatment selectively controls neovascular but not other pathologic components in the OIR mouse model

**Figure 4:**
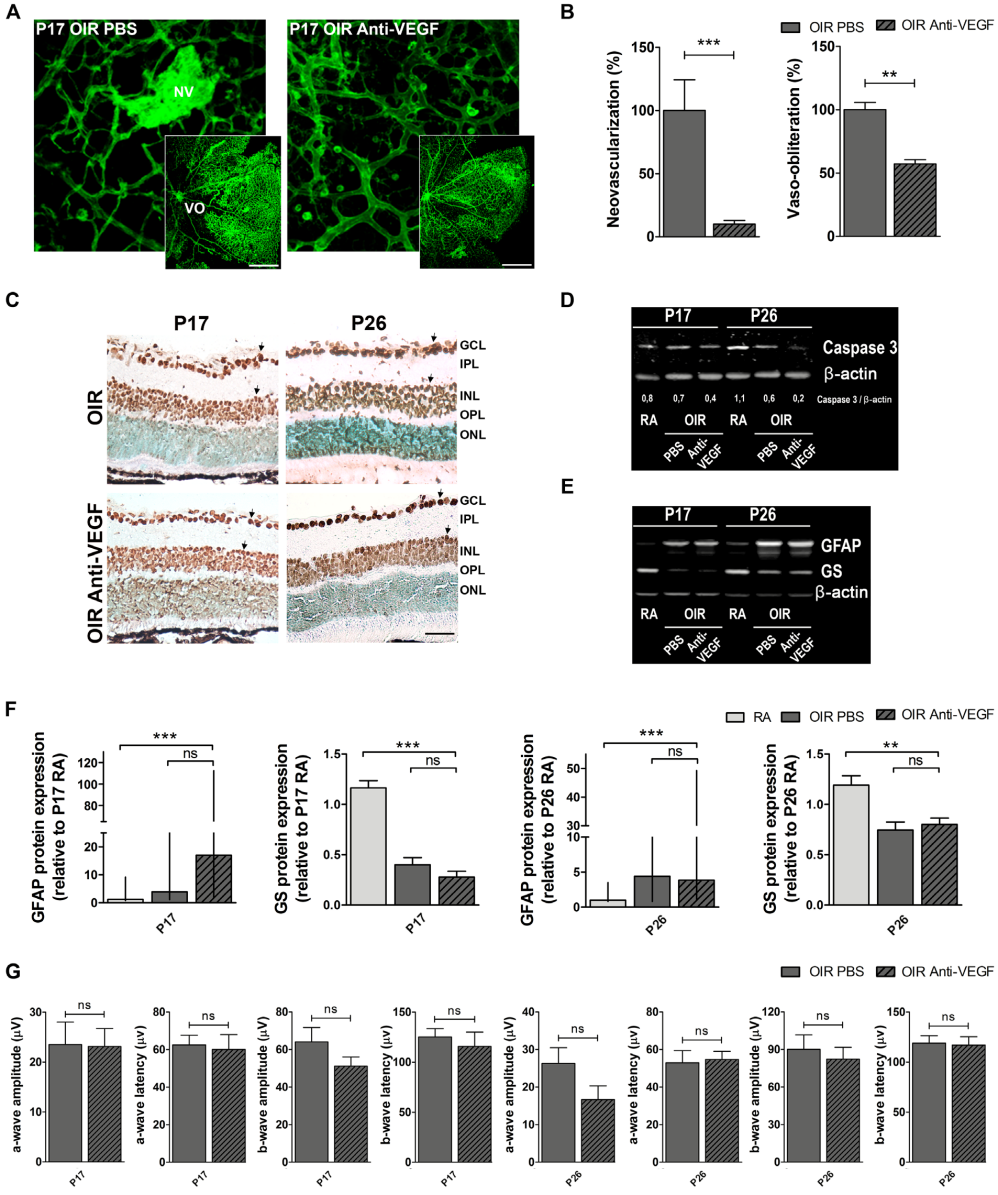
Impact of anti-VEGF treatment in the OIR mouse model **A**. Representative images of whole mount retinas at P17 OIR showing GSA-IB4 vascular staining in PBS (control) or anti-VEGF-injected eyes. Areas with VO and NV are indicated. Scale bar: 100 μm. **B**. The VO (%) was quantified as the ratio of central avascular area to whole retinal area and the NV (%) was quantified as a percentage of whole retinal area. Data are presented as mean ± SEM. **p < 0.01, *** *p* < 0.001. **C**. Representative TUNEL-labeled cryosections of OIR mice injected or not with anti-VEGF at P17 and P26. Scale bar: 50 μm. **D**. and **E**. Representative Western blot of total caspase-3, GFAP and GS from neural retina extracts of RA and OIR mice injected or not with anti-VEGF mAb at P17 and P26. β-actin is shown as a loading control. **F**. Levels of GFAP and GS were quantified by densitometry and normalized to β-actin. Graph shows results of four independent experiments. **G**. Amplitudes and latencies of a- and b-waves from scotopic ERG were recorded at P17 and P26 in OIR mice injected or not with anti-VEGF mAb. Data show the average of responses in both eyes with five mice per condition. Data are presented as mean ± SEM or as median and interquartile range according to parametric or not parametric test used for analysis. ns, non-significant, *p < 0.05, **p < 0.01, *** *p* < 0.001.

A single intraocular injection of anti-VEGF (bevacizumab) administered at P12 in OIR mice improved vascular alterations present in P17 mouse retinas of vehicle-injected OIR eyes (controls) (Figure 4A). Quantitative analysis revealed a decrease of more than 90% and 40% of retinal NV and VO areas, respectively following anti-VEGF treatment (Figure 4B). However, we observed a considerable number of TUNEL-positive cells at P17 and P26 in retinas of OIR control and anti-VEGF-treated mice (Figure 4C). Moreover, we found diminished expression of total caspase-3 in retinal extracts of OIR mice in response to anti-VEGF treatment at P17, an effect that was more pronounced at P26 (Figure 4D), indicating caspase cleavage toward its active form. However, despite attenuation of vascular phenotypes, alterations in GFAP and GS expression were still observed after anti-VEGF treatment (Figure 4E and 4F). In line with these observations, decrease of scotopic electroretinogram retinal response observed in OIR mice (vehicle controls) could not be prevented by anti-VEGF treatment even at P26 (Figure 4G). Of note, no differences in TUNEL staining, GFAP expression and ERG signals were observed between OIR mice injected with vehicle (PBS) or non-injected mice (data not shown). These findings suggest that anti-VEGF therapy does not improve non-vascular alterations associated with NV, including neuronal damage and glial activation and does not amend retinal functional impairment in OIR mice.

### Modulation of the Gal1-glycan axis in OIR mouse retinas in response to anti-VEGF treatment

**Figure 5:**
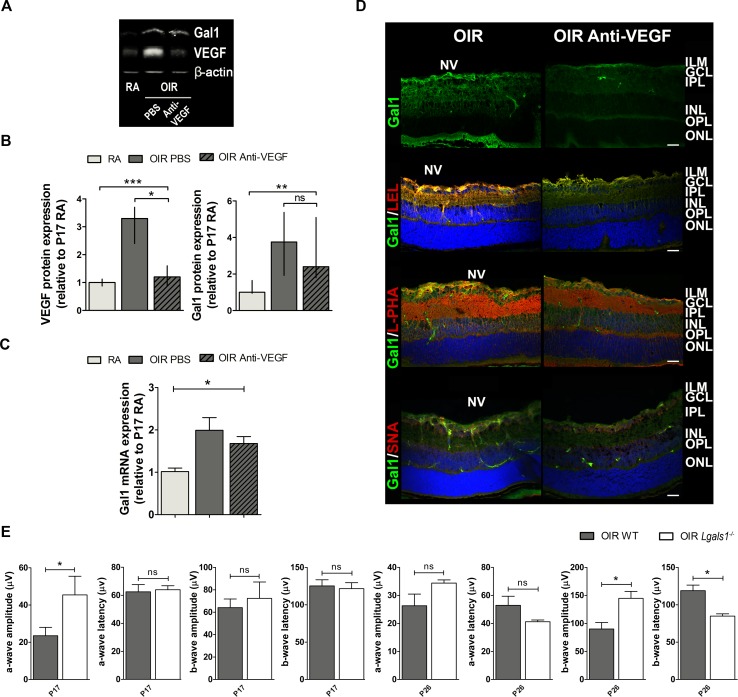
Gal1 expression and function as well as the glycophenotype of mouse OIR retinas after anti-VEGF therapy **A**. Representative Western blot of Gal1 and VEGF from P17 neural retinal extracts of RA and OIR mice injected or not with anti-VEGF mAb. β-actin is shown as a loading control. **B**. Levels of Gal1 and VEGF were quantified by densitometry and normalized to β-actin. Graph shows results of three independent experiments. **C**. Gal1 mRNA levels were quantified by qRT-PCR in neurosensory retinas of P17 OIR mice, injected or not with anti-VEGF mAb, and RA (control) conditions. Results were normalized to β-actin and expressed according to the 2^−ΔΔCt^ method using as calibrator the mRNA level obtained from P17 RA mouse retinas. Data are presented as mean ± SEM or as median and interquartile range according to parametric or not parametric test used for analysis. ns, non-significant, *p < 0.05, **p < 0.01, *** *p* < 0.001. **D**. Representative immunofluorescence analysis of Gal1 (green) or in combination with LEL (red, second panel), L-PHA (red, third panel) or SNA (red, bottom panel), in cryosections of P17 OIR mice injected or not with anti-VEGF. Abbreviations: ILM, inner limiting membrane; GCL, ganglion cell layer; IPL, inner plexiform layer; INL, inner nuclear layer; OPL, outer plexiform layer; ONL, outer nuclear layer; NV, neovascularization. Scale bar: 25 μm. **E**. Amplitudes and latencies of a- and b-waves from scotopic ERG were recorded at P17 and P26 in OIR and *Lgals1*−/− mice. Data show the average of responses in both eyes with three mice per condition. Data are presented as mean ± SEM or as median and interquartile range according to parametric or not parametric test used for analysis. ns, non-significant, *p < 0.05, **p < 0.01, *** *p* < 0.001.

The fact that anti-VEGF treatment reduced the amount of neovascular tufts at P17 but did not improve the associated non-vascular alterations in OIR retinas, prompted us to investigate the impact of VEGF blockade on Gal1 expression and glycan profiles. Results indicated that administration of anti-VEGF at P12 significantly reduced VEGF protein expression in P17, respect to vehicle-injected OIR mouse samples to similar levels to those observed in RA retinas. However, targeting VEGF in OIR mouse retinas did not alter Gal1 expression (Figure 5A and 5B). Moreover, Gal1 mRNA levels were still elevated in retinas of anti-VEGF-treated OIR respect to RA mice (Figure 5C) and localization of this lectin in retinal tissue was not significantly altered in response to this treatment (Figure 5D upper panel). This result suggests that retinal Gal1 expression is independent of VEGF.

To investigate possible alterations in the ‘glycosylation signature’ of OIR mouse retinas following anti-VEGF treatment, we glycophenotyped this tissue using a panel of plant lectins as described in Figure 3. Double fluorescence assays showed colocalization of Gal1 with LEL or L-PHA following VEGF blockade similar to that found in vehicle-injected OIR control retinas (Figure 5D second and third panels). However, because of the absence of NV in anti-VEGF treated mouse retinas, specific colocalization of Gal1 and L-PHA within the vascular compartment was not observed in anti-VEGF treated group (Figure 5D third panel). Furthermore, when we analyzed co-staining of Gal1 and SNA in anti-VEGF-treated retinas, SNA reactivity was similar in both, treated or control retinas at P17 and, as expected, SNA did not colocalize with Gal1. Thus, targeting VEGF does not substantially alter the glycophenotype of retinal tissue in the OIR model.

Notably, functional alterations observed in WT-OIR mice were partially prevented in Gal1-deficient (*Lgals1*−/−) OIR mice, mainly at P26. The scotopic responses resulted in significantly higher b-wave amplitude with a shorter latency in *Lgals1*−/− mice (Figure 5E), demonstrating improved retinal function in OIR mice lacking this endogenous lectin.

### Clinical relevance of Gal1 induction in neovascular retinopathies

**Figure 6:**
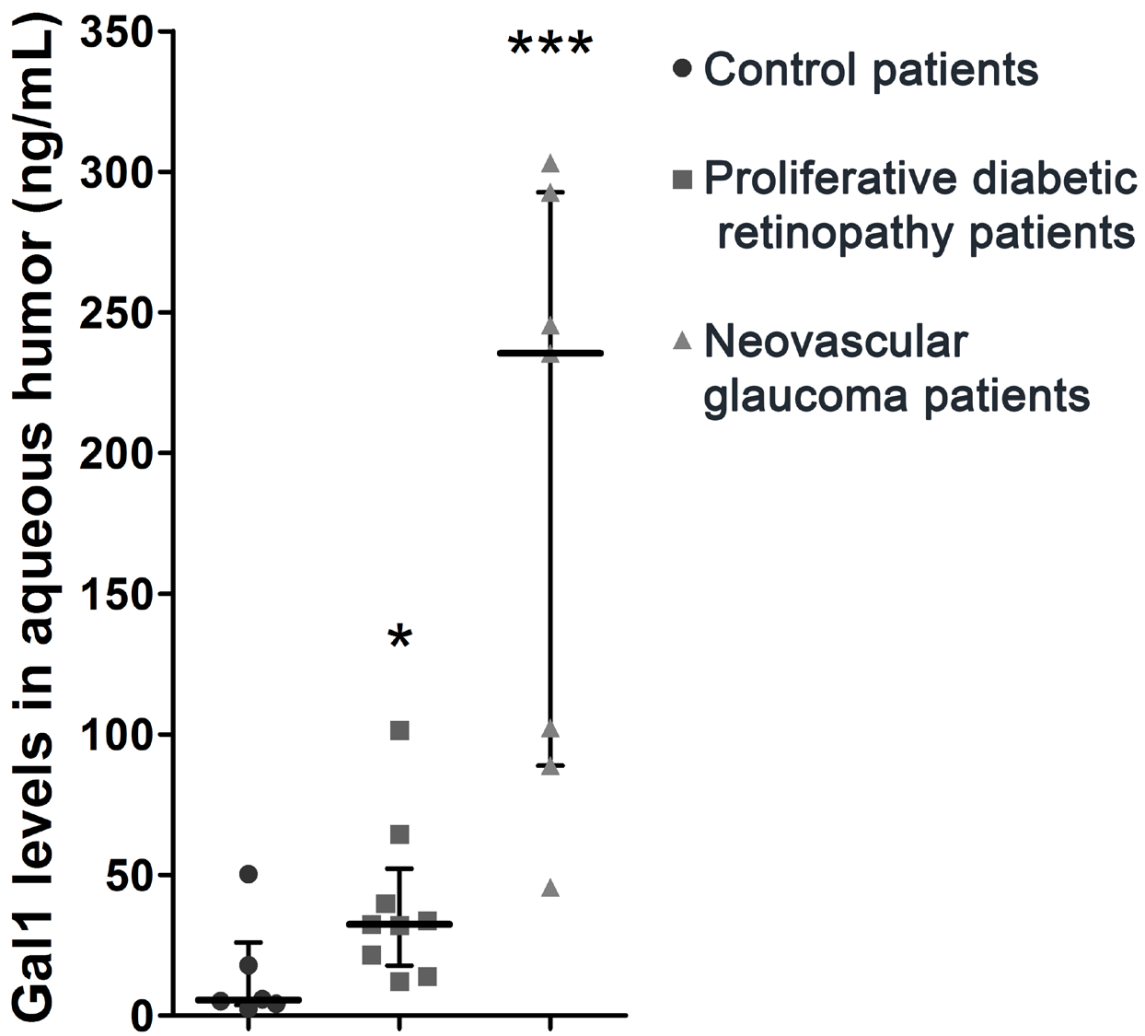
Relevance of Gal1 in human proliferative retinopathies of diabetic patients Representative graph of Gal1 levels in the aqueous humor of control and diabetes mellitus patients with proliferative diabetic retinopathy or neovascular glaucoma. Data are presented as median and interquartile range. ns, non-significant, *p < 0.05, *** *p* < 0.001.

To further extrapolate our results to clinical settings, we next examined Gal1 levels in the aqueous humor of diabetic patients with proliferative diabetic retinopathy or neovascular glaucoma or non-diabetic patients without neovascular retinopathies (controls). As shown in Table 1, we could find no significant differences between groups with regards to age, gender and duration of diabetes mellitus in patients with proliferative diabetic retinopathy and neovascular glaucoma. Interestingly, Gal1 levels increased considerably in proliferative diabetic retinopathy and neovascular glaucoma compared to control samples (Figure 6). Moreover, the highest Gal1 expression was observed in the aqueous humor of neovascular glaucoma respect to proliferative diabetic retinopathy patients. This could be explained by the extent of neovessels proliferation in neovascular glaucoma, an end-stage complication of proliferative diabetic retinopathy. Thus, similar to the experimental OIR model, Gal1 levels are also increased in the aqueous humor of patients with retinal neovascular disorders.

**Table 1:** Clinical features of retinopathy patients

	Cataract (Control group)	Proliferative Diabetic Retinopathy	Neovascular Glaucoma
Number of Samples	6	9	7
Age^a^	70 (60-82) ^ns^	66 (55-74) ^ns^	64 (56-78) ^ns^
Gender (M/W)	4 (67%)/2 (33%) ^ns^	4 (44%)/5 (56%) ^ns^	5 (71%)/2 (29%) ^ns^
Duration of DM^b^	N/A	18 (5-43)^b ns^	15 (10-24)^b ns^

DM, Diabetes Mellitus; N/A, not applicable.

^a^ Age is mean (extreme values) in years.

^b^ Duration of DM is mean (extreme values) in years.

^ns^ Denotes not significantly different.

## DISCUSSION

Pathological retinal angiogenesis is a leading cause of serious vision loss in potentially blinding eye diseases such as proliferative diabetic retinopathy and retinopathy of prematurity [1, 34–36]. Current therapeutic modalities, including “panretinal” laser photocoagulation to reduce retinal and optic nerve head NV, focal photocoagulation and, more recently the intraocular injections of anti-VEGF agents, has sparked a dramatic shift in the treatment of proliferative diabetic retinopathy and diabetic macular edema. However, in most patients the clinical efficacy of these treatments is limited and the complications of diabetes mellitus still represent one of the leading causes of vision loss worldwide.

Previous studies stated that an ideal therapeutic treatment for ischemic retinopathies should combine the normalization of remaining vasculature and the attenuation or prevention of tissue neurodegeneration [34, 36]. It is well known that Müller glial cells and neurons are compromised in experimental neovascular retinopathies, such as in the OIR mouse model [37–39], and that gliosis may contribute to NV and neuronal cell death [40]. Here, we observed an increase of retinal cell death in OIR mice consistent with other studies [41–43] and a persistent induction of GFAP which is expressed in activated Müller glial cells when retinas were injured [12, 44, 45]. In this regard, it is known that persistent gliosis can impair the recycling of neurotransmitters such as glutamate, which accumulation in the retinal milieu causes neurotoxicity [46]. GS, a key neuroprotective enzyme that converts glutamate into glutamine [47], showed decreased expression in both P17 and P26 OIR retinas. Interestingly, and in line with these results, we observed an altered retinal function measured by ERG. The b-wave amplitude (mainly INL cell response [37]) was decreased at P17 and P26 OIR and a- and b-wave latencies were increased at P26 respect to RA mice, which may be attributed to synaptic contacts loss and even neuronal death in the inner retina. Although many studies have previously reported similar alterations in P17 OIR retinas [9, 37, 43], here we detected their persistence until P26 OIR despite vascular normalization.

It was reported that Gal1 is involved in pathological NV and its therapeutic targeting attenuates aberrant angiogenesis in cancer and endometriosis [17, 18, 48–51]; however its role in neovascular retinopathies is still uncertain. In the OIR mouse model, the peak of the proliferative phase (NV) is reached at P17 due to the up regulation of VEGF mostly in glial cells of the inner retina [32]. In addition to increased retinal VEGF at P17 in the OIR mouse model, we demonstrated induction of Gal1 at protein and mRNA levels under hypoxic conditions. Moreover, Gal1 was localized mainly in retinal layers close to the vitreous humor in both OIR and RA conditions; however, it was enriched in areas with high NV in P17 OIR retinas. Immunofluorescence staining suggested its presence in astrocytes, as observed in brain tissue [52] as well as in Müller glial cells similar to expression in chicken retina [53]. In addition, at P17, Gal1 was found inside endothelial cells in neovascular tufts (OIR) but not in normal vessels (RA). Accordingly, we demonstrated here that Gal1 is significantly increased in the aqueous humor of diabetic patients with neovascular retinopathies. Interestingly, patients with neovascular glaucoma exhibited the highest Gal1 levels, an effect that could be associated to the aggressiveness of neovascular proliferation. Interestingly, targeting galectin-3 (Gal3) by 33DFTG, a novel small molecule inhibitor, ameliorates pathological corneal angiogenesis as well as fibrosis [54]. Of note, this inhibitor also binds to Gal1, suggesting that it may control ocular and tumor angiogenesis by inhibiting the effects of both lectins.

In previous studies we showed that interactions between Gal1 and specific target *N*-glycans link tumor hypoxia to NV in Kaposi’s sarcoma through mechanisms involving reactive oxygen species (ROS)-dependent activation of the transcription factor nuclear factor-κB (NF-κB) [17]. Interestingly, ROS have been linked to neovascular retinopathies and antioxidants have been proposed as possible therapeutics agents in proliferative retinopathies [55–57]. Moreover, in OIR conditions and during NV, we observed a glycosylation pattern in retinal tissue permissive to Gal1 binding, as reflected by increased β1-6GlcNAc-branched *N*-glycans and poly-*LacNAc* structures together with reduced α2,6 sialylation compared to RA retinas. Accordingly, it has been reported that the *N*-glycan profile is altered during diabetic retinopathy [58], suggesting involvement of galectin-glycan lattices in these ophthalmologic disorders.

The prominent Gal1 expression in OIR retinas was also maintained at P26 even when retinal vascular alterations were resolved. This observation suggests its involvement not only in retinal NV but also in other processes related to neovascular retinopathies physiopathology. In this regard, the role of Gal1 in neurodegeneration following brain injury has been largely appreciated. It has been reported that Gal1 is involved in the regulation of astrocyte reactivity [59] and that this lectin is important for modulating phagocytosis, inflammation, gliosis and axon growth after spinal cord injury [60, 61]. Moreover, astrocyte-derived Gal1 has been proposed to play a role in neuromodulation and microglia polarization in a model of multiple sclerosis [24] and in axonal degeneration in a model of amyotrophic lateral sclerosis [62]. Recently, it has been observed that Gal1 is upregulated 2-fold in zebrafish retina during experimental degeneration [63], suggesting that it could play an important role in the repair of the zebrafish retina by providing a permissible environment for regeneration. However, since neural retina displays substantial differences with other nervous system circuits, further studies are needed to dissect the role of Gal1 in retinal neurodegeneration in different organisms.

Intravitreal injections of anti-VEGF agents are increasingly used for the treatment of a wide variety of retinal diseases. However, the clinical efficacy of VEGF-targeted therapy is limited and many patients develop resistance to these drugs [64]. Our results demonstrate that anti-VEGF mAb injection at P12 OIR inhibited NV and reduced VO, as previously reported [65, 66], and normalized retinal VEGF levels at P17. However, other pathologic manifestations including gliosis, GS expression decrease and retinal cell death (non-vascular alterations) were preserved in comparison to vehicle-injected OIR retinas. Likewise, altered electroretinogram in OIR mice revealed no significant improvement after intravitreal administration of anti-VEGF agents, indicating that this therapy did not contribute to functional preservation of the neural retina. Notably, deletion of Gal1 significantly improved neuronal function, during retinal ischemia mainly in P26 OIR. These results suggest that, in addition to NV, retinal neurodegeneration should also be considered an important pathogenic component of the disease that could be targeted with appropriate neuroprotective drugs. Our results revealed that Gal1 protein and mRNA increased progressively beyond P17 in OIR even after anti-VEGF treatment demonstrating a neovascular and neurodegenerative role of this lectin in NV-associated retinal dysfunction. In this regard, we recently demonstrated that Gal1 confers resistance to anti-VEGF treatment in different tumor types [30]. Interestingly, tumor-derived Gal1 increased considerably following VEGF blockade, suggesting that spatiotemporal regulation of individual galectins, their selective modulation by hypoxia and the repertoire of glycan structures displayed by tumor vessels, foster the development of a glycosylation-dependent compensatory program that preserves the angiogenic phenotype [30]. These results suggest the possibility of combining VEGF-targeted and Gal1-targeted therapies, as has been proposed with other angioregulatory molecules for the treatment of neovascular retinopathies [48]. In conclusion, our study highlights the importance of non-vascular alterations in proliferative retinopathies and the need of seeking new therapeutic agents targeting both neovascular and neurodegenerative processes to treat this multifactorial disease. Our results underscore the role of Gal1 in the pathogenesis of neovascular retinopathies and suggest potential combination therapies based on simultaneous VEGF and Gal1 blockade in order to attenuate both vascular and non-vascular components.

## MATERIALS AND METHODS

### Animals

WT controls (C57BL/6J) and mice deficient in Gal1 (*Lgals1*−/−) on C57BL/6 background were handled according to guidelines of the ARVO Statement for the Use of Animals in Ophthalmic and Vision Research. *Lgals1*−/−mice were provided by Dr. F. Poirier (Jacques Monod Inst, Paris). Experimental procedures were designed and approved by the Institutional Animal Care and Use Committee (CICUAL) of the Faculty of Chemical Sciences, National University of Córdoba (Res. HCD 451/07). All efforts were made to reduce the number of animals used.

### Oxygen-induced retinopathy (OIR) mouse model

In a typical model of OIR [31], litters of mice pups with their nursing mothers were exposed in an infant incubator to high oxygen concentration (75% ± 2%) for 5 days (hyperoxic period, P7-P12). Oxygen was checked twice daily with an oxygen analyzer (Teledyne Analytical Instruments, CA, USA). Next, mice were housed in RA for an additional time period of 5 or 14 days (relative hypoxic period, P12-P17 or P12-P26). Age-matched control C57BL/6 mice were exposed continuously to RA. Animals were maintained in clear plastic cages with standard light cycles (12 h light/12 h dark).

At P12, some OIR mice were intravitreally injected with 1.0 μL solution containing 1.25 μg anti-VEGF (Bevacizumab; Genentech, San Francisco, CA) as previously reported [66], being representative of the equivalent dose of 1.25 mg anti-VEGF given intravitreally to patients. For 1.25 μg injection, the original commercial solution (25 μg/μL) was diluted 1/20 with phosphate buffered saline (PBS) to obtain 1.25 μg/μL concentration and then 1.0 μL was immediately injected into the vitreous. Vehicle (PBS)/−injected mice were used as controls. Briefly, pups were locally anesthetized with one with one drop of proparacaine hydrochloride 0.5% (Anestalcon, Alcon), exophthalmia was induced with one drop of tropicamide 1% (Midril, Alcon, Buenos Aires, Argentina) and eyes were punctured at the upper nasal limbus as described previously [12].

All mice were sacrificed at three typical times in the OIR mouse model: P12 (maximum VO), P17 (maximum NV) and P26 (vascular alteration resolution) [31]. Eyes or retinas of sacrificed mice were collected and processed for Western blot, real-time PCR (qRT-PCR), immunohistochemistry, immunofluorescence or flat-mount assays. At least six mice per group were used for each condition in the survival times examined. Data were collected from both males and females and the results were combined as there were no apparent sex differences.

### Electroretinography (ERG)

Electroretinographic activity was assessed at P17 and P26 RA, OIR with or without anti-VEGF treatment in WT or *Lgals1*−/−mice, as previously described [37]. Briefly, after overnight (ON) dark adaptation and under dim red illumination, mice were anesthetized *via* intra-peritoneal injections with a solution containing appropriate doses of ketamine / xilacine, the pupils were dilated with 1% tropicamide and the cornea was lubricated with gel drops of 0.4% polyethylene glycol 400 and 0.3% propylene glycol (Systane, Alcon, Buenos Aires, Argentina) to prevent damage. Mice were exposed to stimuli at a distance of 20 cm. A reference electrode was inserted on the back between the ears, a grounding electrode was attached to the tail, and a gold electrode was placed in contact with the central cornea. Electroretinograms were simultaneously recorded from both eyes and ten responses to flashes of unattenuated white light (5 cd.s/m^2^, 0.2 Hz) from a photic stimulator (light-emitting diodes) set at maximum brightness were amplified, filtered (1.5-Hz low-pass filter, 1000 high-pass filter, notch activated) and averaged (Akonic BIO-PC, Argentina).

The a-wave was measured as the difference in amplitude between the recording at onset and trough of the negative deflection, and the b-wave amplitude was measured from the trough of the a-wave to the peak of the b-wave. The latencies of the a- and b-waves were measured from the time of flash presentation to the trough of the a-wave or the peak of the b-wave, respectively. Responses were averaged across the two eyes for each mouse.

### Labeling of flat-mount retinas

Mice were euthanized at P17 and eyes were enucleated and fixed with freshly prepared 4% paraformaldehyde (PFA) for 1-2 h at room temperature (RT). Corneas were removed with scissors along the limbus and the whole retinas were dissected. Then, they were blocked and permeabilized in Tris-buffered saline (TBS) containing 5% Bovine Serum Albumin (BSA) and 0.1% Triton-X-100 during 6 h at 4°C. After that, retinas were incubated ON with 0.02 μg/μL of Isolectin IB4 Alexa fluor-488 conjugate (GSA-IB4) from Molecular Probes, Inc. (Eugene, OR, USA). Retinas were then washed with TBS containing 0.1% Triton-X-100, stored in PBS at 4°C and examined by confocal laser-scanning microscopy (Olympus FluoView FV1200; Olympus Corp., New York, NY, USA).

### Retinal cryosection, protein extract and RNA sample preparation

For cryosection, eyes were enucleated, fixed during 1-2 h with 4% PFA at RT, and incubated ON in 10%, 20% and 30% of sucrose in PBS at 4°C. Then, they were embedded in optimum cutting temperature (OCT) (Tissue-TEK, Sakura) compound, and 10-μm-thick radial sections were obtained by using a cryostat, according to general methods as described [39]. Retinal cryosections were then stored at -20°C under dry conditions until immunohistochemical analysis.

Neural retinas were dissected from RPE/choroid layers for Western blot and qRT-PCR analysis. Protein extracts were obtained from retinas after homogenization with a lysis buffer containing 20 mM Tris-HCl pH 7.5, 137 mM NaCl, 2 mM EDTA pH 8, 1% Nonidet P40, 1 mM phenylmethylsulfonyl fluoride (PMSF), 2 mM sodium ortovanadate and protease inhibitor cocktail (Sigma Aldrich, St. Louis, MO) [39], and were sonicated during 20 sec at 40 % amplitude. In addition, some neural retinas were disrupted in 500 μL Trizol (Invitrogen) and were stored at -80 ˚C until RNA extraction. All the assays were performed in triplicate and results are representative of at least three independent experiments (animals in each group).

### Immunofluorescence and lectin labeling in cryosections

For detection of Gal1 in combination with GFAP, GS and CD31 (PECAM-1) or for LEL, SNA and L-PHA lectin histochemistry, mice cryosections were washed in TBS, blocked with 2% of BSA in TBS containing 0.1% Triton-X-100, for 1 h and then incubated ON at 4°C with the biotinylated lectins (1/200; Vector laboratories) and the following primary antibodies, respectively: mouse monoclonal (1/50) or rabbit polyclonal (1/50) anti-Gal1 obtained as described [30], rabbit polyclonal anti-GFAP (1/100; Dako, Carpinteria, CA), rabbit polyclonal anti-GS (1/100; Abcam Inc., Cambridge, MA) and mouse monoclonal anti-CD31 (1/50; Abcam Inc., Cambridge, MA). Then, sections were washed with TBS 0.1% Triton-X-100 and incubated with Alexa Fluor 555 streptavidin (Thermo Fisher Scientific Inc) or secondary antibodies including goat against rabbit or mouse IgG conjugated with Alexa Fluor 488 and 594 (Molecular Probes, Eugene, OR, USA) respectively, during 1 h at RT. The sections were also counterstained with Hoechst 33258 (1:3000 dilution; Molecular Probes) for 7 min. After a thorough rinse, the sections were mounted with Fluor Save (Calbiochem, La Jolla, CA) and cover slipped. The labeling was visualized using a confocal laser-scanning microscope (Olympus Fluvial FV300 or FV1200; Olympus Corp., New York, NY, USA). Finally, images were processed with microscope software Viewer Fluoview 4.0 software (Olympus) and Image J 2 software (National Institutes of Health, Bethesda, MD, USA). Negative controls without incubation with primary antibody or biotinylated lectin were carried out to avoid unspecific staining (data not shown).

### Western blot

Protein concentration of retinal extracts were determined by a BCA kit (Pierce, Buenos Aires, Argentina) and 10 - 20 μg of proteins were electrophoresed in 15% SDS-PAGE. After electrophoresis, proteins were transferred to nitrocellulose membranes (Amersham Hybond ECL; GE Healthcare Bio-Sciences AB, Uppsala, Sweden). To prevent nonspecific binding, membranes were blocked with 5% milk in TBS containing 0.1% Tween-20 (TBST) during at least 1 h at RT. Then, blots were incubated with primary antibodies diluted in TBST or 5% BSA in TBST for 1 h at RT or ON at 4°C, according to the antibody. The following antibodies were used: rabbit polyclonal (1.5μg/ml; anti-Gal1 generated as described [30]), mouse monoclonal anti-VEGF (1/500; R&D system), rabbit polyclonal anti-GFAP (1/1000; Dako, Carpinteria, CA), mouse monoclonal anti-GS (1/500; Millipore Corporation MA, USA), rabbit polyclonal anti-caspase 3 (Sigma-Aldrich) and mouse monoclonal anti-β-actin (1/2000; Sigma-Aldrich). Blots were incubated with IRDye 800 CW donkey anti-rabbit Ig or IRDye 800 CW donkey anti-mouse IgG antibodies (1/15000 in TBS with 5% BSA) for 1 h, protected from light. After washing with TBST, membranes were visualized and quantified using the Odyssey Infrared Imaging System (LI-COR, Inc., Lincoln, NE, USA).

### qRT-PCR

Total RNA was extracted from neural retinas using Trizol (Invitrogen), according to the manufacturer’s instructions and was processed as previously reported [67]. Briefly, 1 μg of total RNA was reverse-transcribed in a total volume of 20 μL using random primers (Invitrogen, Buenos Aires, Argentina) and 50 U of M-MLV reverse transcriptase (Promega Corp.). For qPCR, cDNA was mixed with 1x SYBR Green PCR Master Mix (Applied Biosystems) and the forward and reverse primers (Gal1 forward: TCAGCCTGGTCAAAGGTGAT/ Gal1 reverse: TGAACCTGGGAAAAGACAGC). qPCR were carried out on an Applied Biosystems 7500 Real-Time PCR System with Sequence Detection Software v1.4. The cycling conditions included a hot start at 95°C for 10 min, followed by 40 cycles at 95°C for 15 sec and 60°C for 1 min. Specificity was verified by melting curve analysis. Results were normalized to β-actin (Forward: GGCTGTATTCCCCTCCATCG/ Reverse: CCAGTTGGTAACAATGCCATGT). Relative gene expression was calculated according to the 2^−ΔΔCt^ method. Each sample was analyzed in triplicate. No amplification was observed in PCRs using as template water or RNA samples incubated without reverse transcriptase during the cDNA synthesis (data not shown).

### TUNEL assay

Cell death was examined by terminal deoxynucleotidyltransferase biotin dUTP nick end labeling (TUNEL) assay (Roche, Mannheim, Germany) following the manufacturer’s instructions. Slides were counterstained with methyl green to visualize retinal layers and then mounted with DPX Mounting Media (Sigma-Aldrich, St. Luis, MO). Negative controls without enzyme were processed in order to avoid false positive results (data not shown). For each section, TUNEL-positive nuclei staining brown were counted from three randomly selected fields on either side of the optic nerve and the average values from three separates sections/eye crossing the optic nerve were combined to produce a mean value. The interval between each section was 15 mm apart. The field size of each picture was approximately 0.05 mm^2^. Images were obtained under a light microscope (Nikon Eclipse TE2000-E, USA).

### Patient samples

The protocol for this study was approved by the Oulton Romagosa Ethics Committee and Córdoba Ministry of Health (Argentina) and the study was conducted according to standards of Helsinki Declaration. Written informed consent was obtained from all participants before their enrollment. Patients older than 21 years old with type-2 diabetes mellitus were recruited between January 2016 and July 2016 from Centro Privado de Ojos Romagosa-Fundación Ver (Cordoba, Argentina). Type 2 diabetes mellitus was diagnosed according to the American Diabetes Association (2005) criteria [68]. Patients were defined as a self-reported previous history of physician-diagnosed type 2 diabetes mellitus treated with insulin or oral hypoglycemic agents and those having a diagnosis of diabetes of at least 5 years were selected. The duration of the disease was defined as the interval between the first diagnosis of diabetes and the time of enrollment in the present study. A questionnaire was conducted to obtain basic information (age & sex), and additional data (including the use of insulin and oral hypoglycemic therapy and any history of other systemic diseases). A complete ophthalmological examination included an external examination of the eye and anexa (including conjunctiva, sclera and lid position or abnormalities), pupil responsiveness (including but not limited to abnormal pupil shape, unequal pupils, abnormal reaction to light or afferent pupillary defect), slit-lamp biomicroscopic examination (cornea, anterior chamber, iris, and lens). Intraocular pressure measurements performed using a Goldmann applanation tonometer performed prior to pupil dilation. The examination also included a dilated indirect ophthalmoscopic examination (including evaluation of the posterior segment and vitreous, optic nerve, retinal vasculature, peripheral retina, and macula) conducted by one of the 2 retinal specialist and dilated fundus photography was done after the complete examination. Seven fields of 30° color fundus photographs with stereoscopic images of the optic disc and macula were taken through the dilated pupils of each patient, using a digital fundus camera (Zeiss Visucam Pro; Oberkochen, Germany). Two retinal specialist ophthalmologists determined the presence and classification of proliferative diabetic retinopathy in a masked manner. All proliferative diabetic retinopathy and neovascular glaucoma patients had, during the last 2 months prior to surgery, different degrees of active neovascularization (disk, arcade or neovascular glaucoma) with or without tractional retinal detachment. Anterior chamber taps were performed under topical anesthesia by microscopic observation of the eye with the patient lying down under a surgical microscope. All this procedure was taken before an intraocular injection of antiangiogenic drug has been applied. By contrast, control group samples were taken before cataract surgery. A drop of 5% povidone iodine was applied to the eye and a limbal paracentesis was performed with a sterile tuberculin syringe with a 30-gauge needle. It was inserted into the anterior chamber, and 0.2 ml of aqueous was removed and stored frozen at -80°C until assayed. Nine samples were collected from diabetic patients with proliferative diabetic retinopathy, seven samples from the patients with neovascular glaucoma, all with end-stage of proliferative diabetic retinopathy, and six samples from non-diabetic patients undergoing cataract were used as a control group as was previously described [69]. Patients with a vitreous hemorrhage were not included in this study.

### ELISA

Gal1 was determined using an in-house ELISA as described [30]. Aqueous humor was previously diluted 1:5. Optical densities were determined at 450-550 nm in a Multiskan MS microplate reader (Thermo Electron Corporation).

### Statistical analysis

Statistical analysis was performed using the GraphPad Prism 5.0 software. A *p*-value < 0.05 was considered statistically significant. Parametric or nonparametric tests were used according to variance homogeneity evaluated by F or Barlett´s tests. Two-tailed unpaired *t* or Mann Whitney tests were used in analysis of two groups. One-way analysis of variance (ANOVA) followed by Dunnett´s multiple comparison post-test or Kruskal-Wallis followed by Dunn´s multiple comparison post-test were used to determine statistical significance among more than two different groups. Two-way ANOVA followed by Bonferroni post-test was used in comparisons between groups when two variables were affecting the dependent variable. Mean ± standard error (SEM) are shown in graphs analyzed with parametric tests and median with interquartile range are shown when data were analyzed with nonparametric tests as indicated in the figures.
